# Supplementary figures and images for: Emergence of an early SARS-CoV-2 epidemic in the United States
Source: medRxiv. 2021 Feb 8:2021.02.05.21251235. Preprint. [Version 1] doi: 10.1101/2021.02.05.21251235 (PMC7872376; doi:10.1101/2021.02.05.21251235)

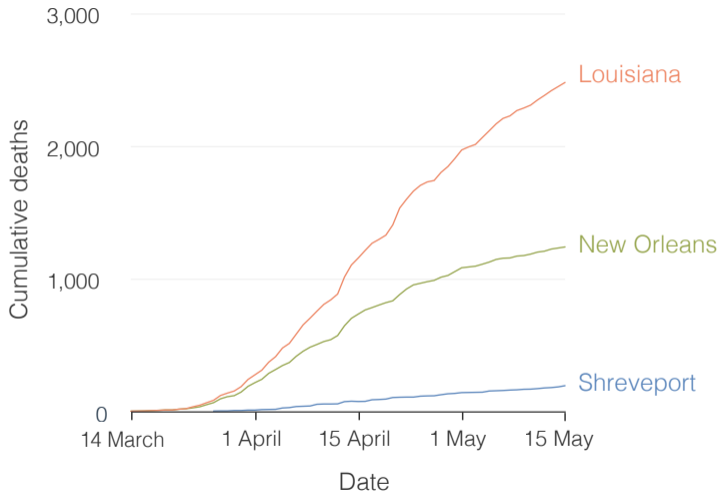

Supplement: Supplement 1 — Figure S1. Cumulative SARS-CoV-2 death during the first wave of the COVID-19 epidemic in Louisiana. https://bit.ly/2MF5u5x [file media-1.pdf]

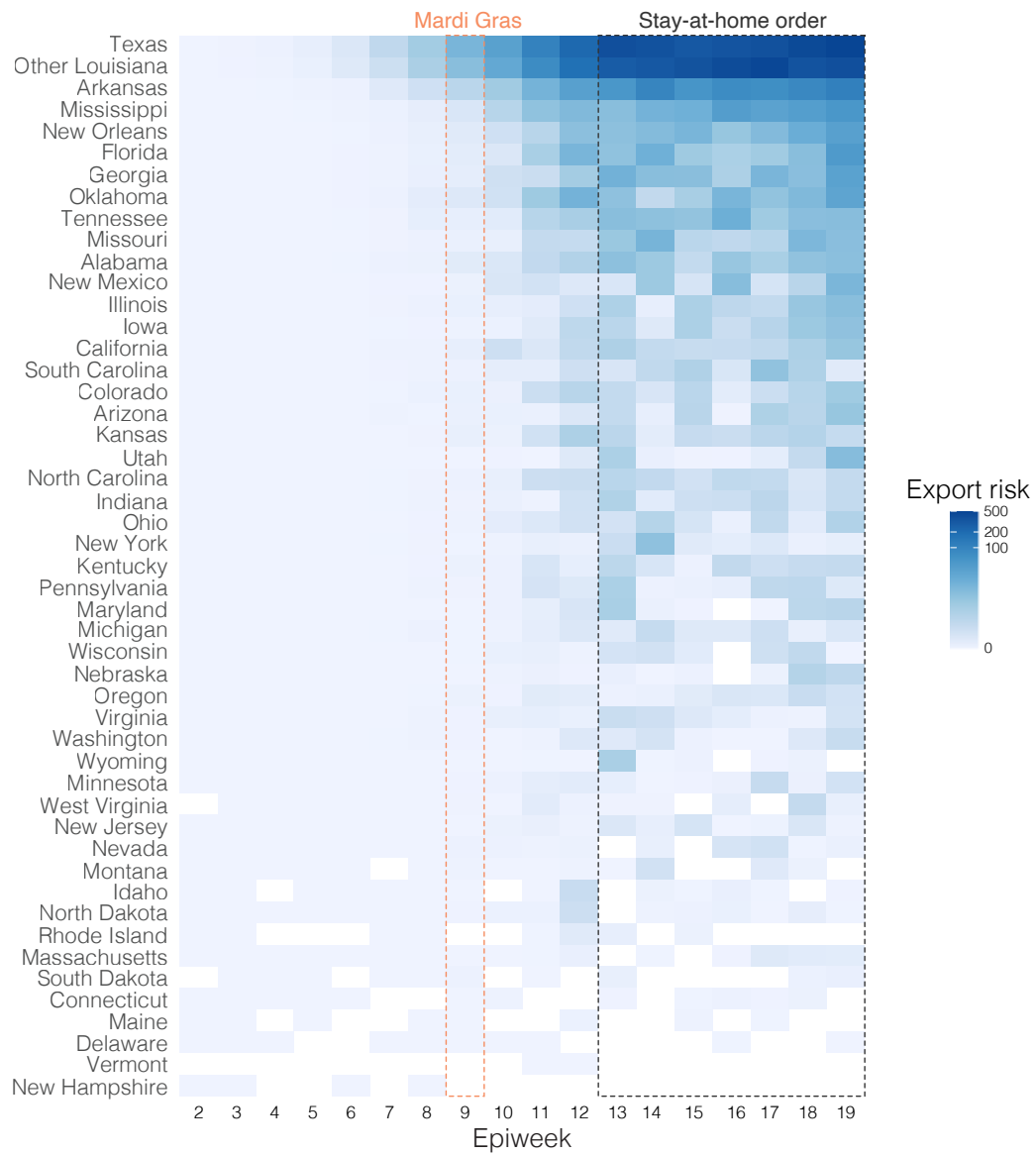

Supplement: Supplement 2 — Figure S2. Estimated number of travelers from Shreveport per epiweek. https://bit.ly/2YMZIRN [file media-2.pdf]

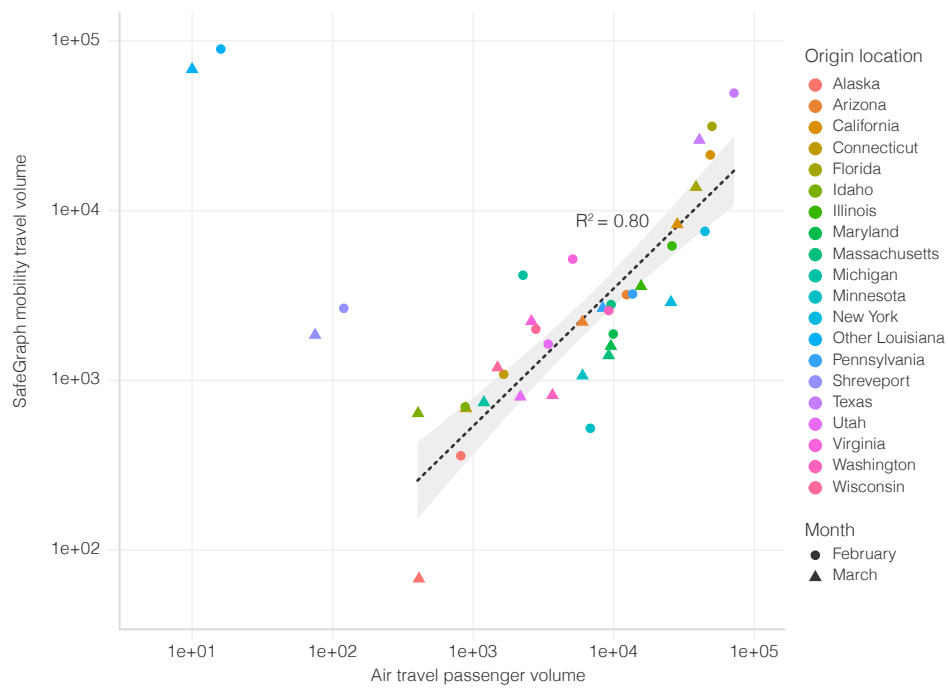

Supplement: Supplement 5 — Figure S5. Correlation between travel datasets. Air travel passenger volumes and SafeGraph mobility travel volumes from various U.S. states into New Orleans. Spearman rank correlation does not include Shreveport and Other Louisiana, since air travel is not the dominant mode of transport to New Orleans for these locations. https://bit.ly/2YOA7If [file media-5.pdf]
